# Comparative analysis of protein coding sequences from human, mouse and the domesticated pig

**DOI:** 10.1186/1741-7007-3-2

**Published:** 2005-01-28

**Authors:** Frank Grønlund Jørgensen, Asger Hobolth, Henrik Hornshøj, Christian Bendixen, Merete Fredholm, Mikkel Heide Schierup

**Affiliations:** 1 Department of Ecology and Genetics, University of Aarhus, Aarhus C, Denmark; 2 Bioinformatics Research Center (BiRC), University of Aarhus, Arhus C, Denmark; 3 Department of Genetics and Biotechnology, Danish Institute of Agricultural Sciences, Tjele, Denmark; 4 Department of Animal Science and Animal Health, KVL, Frederiksberg C, Denmark

## Abstract

**Background:**

The availability of abundant sequence data from key model organisms has made large scale studies of molecular evolution an exciting possibility. Here we use full length cDNA alignments comprising more than 700,000 nucleotides from human, mouse, pig and the Japanese pufferfish *Fugu rubrices *in order to investigate 1) the relationships between three major lineages of mammals: rodents, artiodactyls and primates, and 2) the rate of evolution and the occurrence of positive Darwinian selection using codon based models of sequence evolution.

**Results:**

We provide evidence that the evolutionary splits among primates, rodents and artiodactyls happened shortly after each other, with most gene trees favouring a topology with rodents as outgroup to primates and artiodactyls. Using an unrooted topology of the three mammalian species we show that since their diversification, the pig and mouse lineages have on average experienced 1.44 and 2.86 times as many synonymous substitutions as humans, respectively, whereas the rates of non-synonymous substitutions are more similar. The analysis shows the highest average dN/dS ratio in the human lineage, followed by the pig and then the mouse lineages. Using codon based models we detect signals of positive Darwinian selection in approximately 5.3%, 4.9% and 6.0% of the genes on the human, pig and mouse lineages respectively. Approximately 16.8% of all the genes studied here are not currently annotated as functional genes in humans. Our analyses indicate that a large fraction of these genes may have lost their function quite recently or may still be functional genes in some or all of the three mammalian species.

**Conclusions:**

We present a comparative analysis of protein coding genes from three major mammalian lineages. Our study demonstrates the usefulness of codon-based likelihood models in detecting selection and it illustrates the value of sequencing organisms at different phylogenetic distances for comparative studies.

## Background

Large scale sequencing projects of many different species allow us to investigate phylogenetic issues in much more detail and to identify whether certain genes have had an extraordinary evolution in one or more species and thus gain insight into the actions of natural selection. Despite the sequencing of an increasing number of mammalian genomes and the implementation of more sophisticated evolutionary models using maximum likelihood and Bayesian methodology, the branching order within the mammalian phylum is still not completely resolved. The main reason for this uncertainty is that the diversification of these orders occurred over a short period of time, making the inference of branching order a difficult problem. One of the highly debated issues concerns the relative order of branching among primates, artiodactyls and rodents [1-9]. Here, the Japanese pufferfish *Fugu rubrices *is used as an outgroup to estimate the branching order of the three species relative to each other.

Codon based models [10,11] allow for powerful analysis of protein coding nucleotide sequences. Evolutionary hypotheses may be tested using likelihood ratio tests between nested models. For an introduction to the practical use of these models see [12], for a more thorough review of the methodology see [13]. The parameter of primary interest is the ratio of nonsynonymous to synonymous substitutions (ω), also known as the dN/dS ratio. The dN/dS ratio measures the relative importance of evolutionary forces that have shaped a particular protein. A dN/dS ratio significantly larger than one strongly suggests that positive Darwinian selection has acted on the protein. Different extensions to the basic codon model exist, and these can be divided into three main categories: (1) Lineage-specific models that average ω over sites but differentiate between lineages [14]; (2) site-specific models that average ω over lineages but differentiate over sites [15]; (3) branch-site specific models that combine the two previous extensions by allowing ω to vary over sites in all background lineages, but allow for a different value of ω in one or more pre-specified lineages [16]. The models we use here and their relationships are shown in Table 1. Numerous studies have shown the ability of the site-specific and the branch-site specific models to detect positive selection in cases where the branch-specific models did not, indicating that averaging over sites is generally a more serious problem than averaging over lineages and that in many cases using a branch-site specific model increases the power to detect positive selection [17-22].

**Figure 1:**
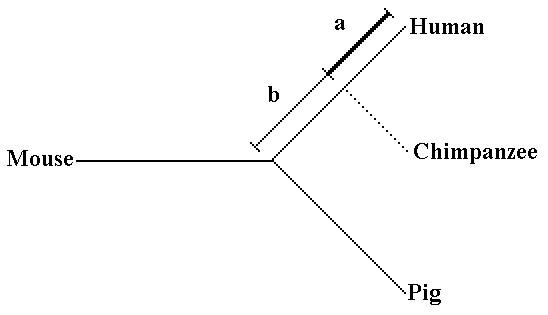
Phylogenetic tree of key mammalian species. A schematic drawing showing the topologies considered in our study compared to a recent study on human, chimpanzee and mouse trios [23]. Branch a shows the branch considered in the study by Clark et al (2003) while branch a+b represents the evolutionary time scale studied here.

In a recent study of cDNA trios of human, mouse and chimpanzee a codon based branch-site specific model was used to search for human genes that have undergone positive selection since our divergence from other primates [23]. Here, a similar search is done on a different phylogenetic level using a collection of porcine genes. While the study by Clark and colleagues concentrates on the divergence between humans and chimpanzees (branch **a **in Figure 1) our study searches for genes that have undergone positive selection since the divergence of primates, artiodactyls and rodents. Several recent studies have shown that some of the branch-site specific models under certain conditions might have a high false positive rate when used to detect positively selected sites [24,25]. This problem has recently been addressed by Yang and colleagues with the implementation of a new Bayes empirical Bayes (BEB) method for predicting positively selected sites. This new method is much better at avoiding false positives while still retaining a high sensitivity (Z. Yang, pers. comm.). Here we use the new and improved BEB version of the branch-site specific model originally presented in [23] to detect genes that may have been influenced by positive selection.

## Results

**Figure 2:**
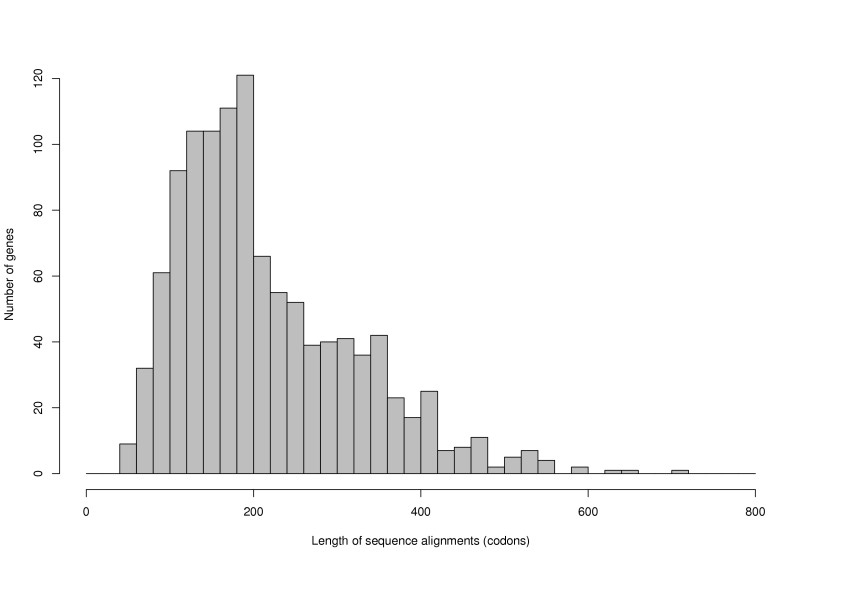
Distribution of sequence alignment lengths. Histogram showing the distribution of sequence lengths in the three species alignments.

The distribution of sequence lengths of the 1120 three-species alignments is shown in Figure 2. Since the full length cDNAs were assembled from random ESTs, there is a bias towards assembling relatively short genes. Therefore the subset of genes used in this analysis is not a random sample from the pig genome. This decreases the power of our evolutionary tests, since short alignments have less power when testing for positive selection, but we do not anticipate any other systematic bias in our results.

### Mammalian phylogeny

**Figure 3:**
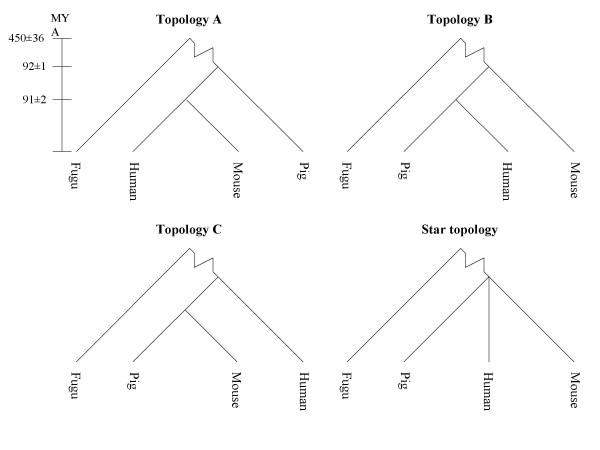
Conflicting mammalian phylogenies. A schematic drawing of the three conflicting bifurcating topologies (a-c) as well as a multifurcating alternative (d). The divergence times shown in (a) are million years from present [31].

The relative branching order of the three mammalian species was investigated with the individual genes as well as with a concatenated super gene. Using the empirical amino acid substitution model of Whelan and Goldman [26] we maximized the likelihood under the three conflicting topologies shown in Figure 3a–c. In 123 of the 988 alignments all amino acids are identical in the three mammalian species giving us no information to discriminate between the three topologies. Of the remaining 865 alignments 245 favour topology A, while 440 and 180 favour topology B and topology C respectively. A concatenated super gene of all 988 alignments clearly favoured topology B over topology A, which again has a higher likelihood than topology C, consistent with the results from the individual gene comparisons (Table 2.).

We used the baseml program of PAML to compare the three topologies in a nucleotide based framework. Different nucleotide based substitution models were used to maximize the likelihood on the three topologies for each of the three codon positions separately. The results of using different models of nucleotide evolution were highly similar so here we only discuss the results obtained with the HKY85 model [27]. The results based on the third codon position shows that Fugu is too distantly related to the three mammals to be informative in placement of the root of the mammals (results not shown). The first and second codon positions do not show such saturation and should therefore be useful in comparing the three topologies. Consistent with the results based on the amino acid substitution model we see that topology B is favoured in most genes, followed by topology A and topology C, respectively. The actual numbers from the second codon position are 215, 386 and 179 in favour of topology A, topology B and topology C respectively and 208 alignments are uninformative. The corresponding numbers for the first codon position are 215, 545, 175 and 53 (Table 2.).

The internal branch is rather short in all cases. Therefore in the remaining analyses we treat the mouse, human, pig split as a trifurcation. Depending on which topology is actually the right one, the only bias introduced by treating the topology as a star tree, as shown in Figure 3d, is a minor overestimation of the branch length of the species that actually roots the other two.

### The rates of evolution

**Figure 4:**
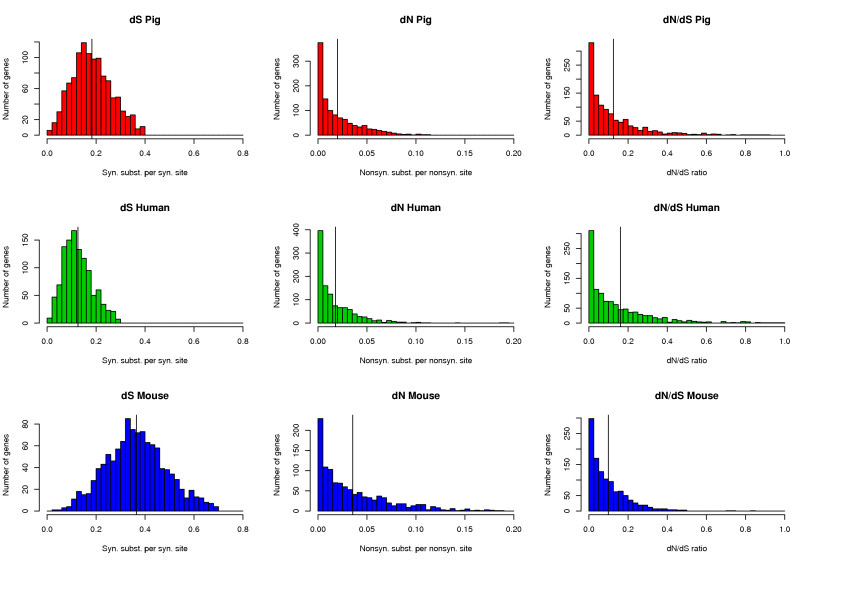
Evolutionary rates. Histograms of key parameters in the codon models. (a-c) The rate of synonymous substitutions per synonymous site (dS) in the pig, human and mouse lineage respectively. (d-f) The rate of nonsynonymous substitutions per nonsynonymous site (dN) in the pig, human and mouse lineage respectively. (g-h) The ratio of nonsynonymous substitutions to synonymous substitutions (dN/dS ratio) in the pig, human and mouse lineage respectively. The horizontal line represents the mean of the distributions.

The three-species alignments were used to estimate the synonymous and nonsynonymous substitution rates of the three branches under the free ratio model, see Table 3. Figure 4a–f shows the distribution of the synonymous and nonsynonymous branch lengths for each gene in all three species. The synonymous rates are significantly different between the three species. The average synonymous substitution rate, estimated using the concatenated super gene, is approximately 2.86 times larger in mouse compared to pig, and approximately 1.44 times larger in pig than in human. The nonsynonymous rates are more similar among the three species. The corresponding values for the nonsynonymous rates are 2.08 and 1.17 respectively. Table 3 shows the mean, median and variance of both the synonymous and nonsynonymous rate distributions as well as the values obtained from the concatenated super gene. The average values from the individual genes are highly similar to the results obtained from the concatenated super gene.

### Positive Darwinian selection

The dN/dS ratios on the three different lineages were estimated under the free ratio model (Figure 4g–i). Most genes in all three species have an average dN/dS ratio very close to zero with the average dN/dS ratio higher in human than in pig, which again is higher than in the mouse lineage.

The one ratio model averages over sites and lineages, which makes this an extremely conservative method of detecting positive selection. Only four of the 1120 three-species alignments have an average dN/dS ratio larger than one, see Table 4, and of those only one is significantly larger than one (XM_165930). The free ratio model allows each lineage to have its own dN/dS ratio. This model has slightly more power than the one ratio model due to its ability to find lineage specific signals. The likelihood ratio test (LRT) of these two models should not be considered as a stringent test for positive selection, but more as a test for different selective forces among lineages. The LRT shows that 154 genes have significantly different dN/dS ratios among lineages at the 5% significance level, 73 at 1% and 41 at the 0.1% level of significance. Table 5 shows the 24 genes that have a dN/dS ratio larger than one in one or more lineages as well as the result from each gene of a LRT that tests whether the estimated value of ω is significantly larger than one. As with the one ratio model only one gene shows a result significantly larger than one. The gene is the same one as reported with the one ratio model (XM_165930) and the lineage with a dN/dS ratio significantly larger than one is the lineage leading to pig.

Several studies have shown that averaging over sites is more conservative when searching for positive selection than is averaging over lineages. The branch-site specific model A and model B [16] were originally designed to search for genes where only a small fraction of codons in a specific foreground lineage has evolved under positive selection. Several studies have shown that the original models are prone to predicting false positives under certain conditions, and one should therefore be very careful drawing conclusions from studies based on those models. Here we use a new and improved version of a branch-site model developed for the analyses of human, chimpanzee and mouse gene trios [23]. The new model we use here is implemented in PAML v. 3.14 and uses the new and improved Bayes empirical Bayes approach to predict which sites have evolved under positive selection in the foreground lineage. Likelihood ratio tests were done separately with human, pig and mouse as the predefined foreground lineage. The LRT when contrasting the neutral model with the branch-site model has two degrees of freedom. By using the human lineage as foreground lineage we find 288 genes that show signals of positive selection (dN/dS in the foreground lineage is larger than one). In 58 of those genes the branch-site model fits the data significantly better than the neutral model at the 5% significance level. We find 34 and 15 genes at the 0.01 and 0.001 levels of significance respectively. The corresponding numbers of genes using pig as foreground lineage are 314, 55(0.05), 23(0.01) and 5(0.001). Using mouse as foreground lineage results in 352, 67(0.05), 25(0.01) and 4(0.001). The genes found to be under positive selection in any of the three species with a LRT significance level of 0.001 are shown in Table 6.

The molecular function of the genes predicted to be under positive selection was determined using the Panther server [28] and the NCBI server using the newest build of the human genome. Both annotation servers are updated on a regular basis when new information becomes available. During the course of this study the annotation of several genes changed. Of our 1120 alignments 188 are currently not annotated as functional genes indicating that they might possibly be pseudogenes in human; see the Discussion for more details on this subject. The proportion of genes that we report to have undergone positive selection in the human lineage at the 5% level of significance can therefore be viewed as either 58/1120 ~5.2% or 43/931 ~4.6%, indicating that possible pseudogenes are only slightly overrepresented in the genes predicted to have undergone adaptive evolution. The genes predicted to have been under positive selection in the pig and mouse lineage show a similar trend.

Several different models have been developed that allow for heterogeneity of ω over sites in an alignment. We used the M4 model [15] which allows each codon to fall into one of 5 categories corresponding to ω equal to 0, 1/3, 2/3, 1 and 3. The first category represents the fraction of codons that have evolved under strong purifying selection allowing no nonsynonymous changes to occur. The next two categories represent different intensities of purifying selection. The category with ω = 1 represents neutrally evolving sites, while the last category with ω = 3 represents codons that have evolved under positive selection. The results of this analysis on the concatenated super gene can be seen in Table 7. Only 1.6 % of all codons appear to have evolved under positive selection, and approximately 69 % have been under strong functional constraints.

### Codon usage bias

The concatenated super gene was also used to investigate the patterns of codon usage in the three species; the results of this investigation are summarized in Table 8. A test for equal codon distributions is rejected in all three pair wise comparisons (P < 0.0001, 60 d.f.). Using nucleotide frequencies to estimate the codon equilibrium frequencies fits the data poorly, so does the equal frequency model (Table 9). For a description of the codon equilibrium frequency models, see the Methods. The F3 × 4 model was extended with one extra parameter that accounts for CpG avoidance at the second and third codon position. Since all changes in the second position of a codon are nonsynonymous, the frequency of NCG codons is expected to be lower than under the F3 × 4 model. The extra parameter introduced improves the log likelihood by approximately 1236 units (~44%). This can be compared to the approximately 321 units per extra parameter introduced when going from the F3 × 4 model to the codon table model. When analysing the super gene it is still better to use the actual codon frequencies, but with individual genes the number of codons can sometimes be so small that the use of actual codon counts can be problematic. We also implemented a similar model that incorporated the avoidance of CG in first and second position by introducing an additional parameter but this does not improve the fit of the model significantly (results not shown). This is probably caused by the fact that all four codons with CG in the first and second position code for the same amino acid, Arginine. Arginine has six different codons and the two codons without a CG pair (AGA and AGG) are generally favoured over the other four (Table 8), but this tendency is apparently accounted for when modelling nucleotide frequencies at the three codon positions, so here we only present the model that accounts for CpG avoidance at the second and third codon position. Table 9 shows that the choice of codon equilibrium frequency model has detectable effects on the parameter estimates. Most striking is the apparent overestimation of the transition/transversion ratio and the dN/dS ratio when the model is less parameter-rich.

## Discussion

The phylogeny of the early mammalian radiation has been extensively debated over the last two decades. The classical view based on fossil evidence states that all major orders of placental mammals first appear right after the Cretaceous-Tertiary (KT) boundary approximately 65 million years ago [29]. This sudden appearance of all major placental orders is known as the mammalian radiation. With the use of molecular data this late radiation has been challenged and it is now widely accepted that the radiation of the placental orders probably occurred many million years before the KT boundary [29-31]. Molecular data have also been used to investigate the relative branching orders of many of the larger clades of placental mammals [1-7,9,30]. One of the issues that have been debated extensively is the placement of Rodentia in the placental tree. Some studies favour a basal placement of the rodents [1,3-5,32,33] while other studies favour a sister relationship between primates and rodents [6-8]. Recently strong evidence based on insertions, deletions and ancient transposable elements in favour of a sister relationship of primates and rodents has been reported [2,34].

The incongruence of single gene phylogenies was investigated in a recent study of eight yeast species [35]. The phylogeny commonly believed to be correct is completely resolved when concatenating 20 or more randomly chosen genes to form a super gene. A concatenated multi gene approach was also shown to resolve single gene incongruences in a recent study on green algae [36]. Here we use 988 full cDNA alignments comprising 672,918 nucleotides to investigate the branching order of the three mammalian species. We present results based on both single gene phylogenies and a concatenated super gene. All genes including the concatenated super gene were analysed with both nucleotide and amino acid based substitution models. All methods favour a primate-artiodactyls clade with rodents as an outgroup but with a relatively short internal mammalian branch, indicating that the mammalian radiation happened within a short period of time. The different methods used in this study have very different assumptions but they all show the same general results. The HKY85 model takes into account differences in nucleotide frequencies and transition/transversion biases and allows for differences in substitution rates among the lineages. However, it is still possible that complexities unaccounted for such as non-stationarity and irreversibility of the substitution process have created biases that lead to long-branch attraction of Fugu and Mouse and an erroneous conclusion. Furthermore, the incongruence between our analysis and many recent studies is also affected by the following. (1) The choice of outgroup; bony fishes are believed to have diverged approximately 450 million years ago [31], making saturation effects in synonymous sites a real problem. We are therefore forced to only consider nonsynonymous sites or amino acid replacements in the phylogenetic analyses. The recently completed genome sequence of the chicken (*Gallus gallus*) shows that the average value of dS between human and chicken genes is approximately 1.66 [37], which indicates that many genes may still be too distantly related for synonymous sites to avoid problems with saturation. A marsupial species would provide a much better outgroup when available [3,32]. (2) Taxon sampling; by only using three species the variance of the parameter estimates can be quite high and the power to discriminate between two conflicting topologies quite low. The sequencing of more species will lessen this problem. (3) Overly simplistic evolutionary models; here we use only nucleotide and amino acid based models. If a more closely related outgroup was available the use of more complex codon based models could be beneficial in resolving the apparent conflict. Several extensions have been made to the codon models during the past few years. One obvious extension to the codon models is a model that incorporates CG avoidance within and over codon boundaries. This will clearly improve the fit of the data to the model and therefore give more accurate parameter estimates. Including context dependencies over codon boundaries and information about protein structure have also been shown to increase the fit of the models to protein coding data and therefore should result in better parameter estimates [38,39]. (4) Gene trees and species trees can be different; the split between the three groups probably occurred within a very short period of time, allowing for the possibility that different genes actually have different phylogenies due to ancient polymorphisms at the time of the speciation. Using even larger number of genes and a sufficiently sophisticated model should lessen this problem [35,36].

The rate of synonymous substitution was estimated to be almost three times higher in rodents than in other mammals, in agreement with previous investigations that also showed an elevated rate in rodents [40-42]. This has historically often been explained by a generation time effect. Species that have short generation times experience more generations in the time span we consider and consequently they will experience more neutral substitutions over time. The fact that the pig, which has a generation time intermediate between mouse and humans, has an intermediate rate of synonymous substitutions, seems to agree with this theory. For a more thorough discussion of the generation time hypothesis in mammals see [43]. The nearly neutral theory of molecular evolution predicts that the generation time effect should be smaller for non-synonymous substitutions [42,44,45]. The simple argument is that animals with short generation times such as rodents often have a very large effective population size. In a population with a large effective population size slightly deleterious mutations will be removed from the gene pool more effectively than in a population with a small effective population size, where genetic drift will reduce the efficiency of natural selection. Figure 4g–h shows the distribution of the dN/dS ratio in the three lineages. The average dN/dS ratio is highest in humans suggesting a small effective population size, while it is smallest in mouse suggesting a larger effective population size.

Previous studies of the occurrence of positive selection based on pair wise comparisons have revealed a very low occurrence of positive selection. In a study of 3595 alignments only 17 genes showed evidence of positive selection [46]. The branch specific models used here only find one gene where the dN/dS ratio is significantly larger than one. The gene reported is XM_165930. XM_165930 was originally annotated as being similar to cold shock domain protein A, but it has recently been removed from Genbank as a result of standard genome annotation processes.

Codon based branch-site models similar to the ones used here were used in a paper based on a three way comparison among chimpanzees, humans and mice [23]. They report that approximately 1.6 % of all the genes studied have been undergoing positive selection in the lineage leading to modern humans. Using a similar criterion our study indicates that approximately 3.0 % of the genes studied have been undergoing positive selection on the lineage leading to humans; the corresponding numbers for pig and mouse are 2.0 % and 2.2 % respectively. When comparing these two studies it is important to consider the following three things: (1) the relatively short average length of the genes studied here decreases the power of the models to detect positive selection; (2) the use of the new BEB method for detecting positively selected sites should reduce the number of false positives, making our estimates more conservative and more accurate; (3) our study deals with a completely different phylogenetic level, covering a much longer time span than the study by Clark and colleagues.

The multiple testing and the small number of taxa used in a study like this imply that the results presented should not be taken as conclusive evidence for positive selection, but more as an approach to searching among the thousands of genes to look for genes that may have evolved in a biologically interesting manner. Comparative approaches such as the one we use here can only be a first step towards showing that positive Darwinian selection may be a key part in the evolution of many different gene families. Further experimental and computational analyses must then be used to investigate the suggested candidates more thoroughly.

During the course of our investigation a large fraction of the genes were re-annotated as putative pseudogenes: 188/1120 ~16.8%. However, all these genes have uninterrupted reading frames in all three species; only a tiny fraction of all codons seems to have evolved in a neutral-like fashion (ω~1), and the distributions of the synonymous as well as the nonsynonymous rates of these putative pseudogenes are almost identical to the distributions of the remaining genes (results not shown). The only difference is a slight increase in the dN/dS ratio in the human lineage, which is actually due to a few genes that experience an unusually high dN/dS ratio. Omitting these genes from the analysis removes the observed differences completely. Thus, if all these genes are indeed pseudogenes in human, the loss of function must have occurred quite recently and they may not be pseudogenes in pig and mouse.

## Conclusions

The collection of a large set of pig cDNA sequences has enabled us to study long term evolutionary trends in mammalian genes. Our results indicate that the codon models are able to detect evolutionary signals indicating adaptive evolution in several genes. Our phylogenetic investigation of the primate, rodent, artiodactyl split disagree with most recent findings in favouring a primate, artiodactyl clade with rodents as an outgroup. Our study indicates that several genes that are not classified as genes in the most recent human annotation might after all be real genes; or at least they have become pseudogenes very recently, and the orthologous genes in mouse and pig might still be functional. This shows the potential of comparative methods in identifying functional regions of the genome.

## Methods

### cDNA alignment

Complete cDNA from the domesticated pig *Sus scrofa *was assembled at the Danish Institute of Agricultural Sciences (DIAS) from cDNA libraries from 100 different tissues constructed at DIAS and the Royal Veterinary and Agricultural University in the following way. Total RNA was purified from selected tissues using Rneasy (Qiagen) or Tri ReagentR and poly(A+) mRNA was selected using Oligotex (Qiagene) or PolyATract (Promega). Directional cloneable cDNA was synthezised from Poly(A+) mRNA using the cDNA Synthesis Kit (Stratagene) and was ligated into Eco RI/Xho I digested pTrueBlue (GenomicsOne) or pBluescript (Stratagene) followed by electrotransformation into E. coli XL1-Blue MRF' (Stratagene). 5'-EST sequencing was performed using standard protocols (Applied Biosystem). The sequences were trimmed to the longest open reading frame and the termination codons were removed.

Homologues sequences from human, mouse and the Japanese pufferfish *Fugu rubrices *were obtained with the blastall program with default parameters; the E-score was set to 10^-8^. We constructed two different datasets, one with and one without *Fugu rubrices*. Individual alignments were made using ClustalW version 1.83 with default parameters [47]. We kept the pig reading frame intact in the alignments by removing any columns where the alignment gave rise to gaps in the pig sequence. Alignments that resulted in premature stop codons, or were shorter than 30 codons, were removed. We used the one ratio model to estimate the total branch length of the tree as well as the synonymous branch lengths. These distributions were used to detect peculiar genes where one or more sequences might not be a true orthologue, and all outliers were thereafter removed from the dataset. This analysis gave 1120 alignments of mouse, human and pig, and of these 988 also included Fugu. The 1120 original cDNAs from *Sus scrofa *have been deposited in Genbank with the following accession numbers: AY609387-AY610506.

### Phylogeny and rates of evolution

Nine hundred and eighty-eight four-species alignments were concatenated into a super gene. The three topologies were compared using the super gene as well as each individual gene. Both nonsynonymous nucleotide substitutions and amino acid substitutions were investigated with PAML v. 3.14 [48]. The nonsynonymous substitutions were represented by the first and second codon positions of all codons, and the three different topologies were investigated with baseml using the HKY85[27] model (model = 4) of nucleotide substitutions. The likelihood was then maximized under the three different topologies using all the individual genes as well the concatenated super gene. The codeml program with the codons translated to amino acids (seqtype = 3) were also used to investigate the three topologies. We used different models of amino acid evolution to maximize the likelihood under the three topologies and since the results were highly similar we only present the results from the empirical method of Whelan and Goldman (model = 2, aaratefile = wag.dat)[26].

Using the 1120 three species alignments, the synonymous and nonsynonymous rates of evolution were estimated with the codeml program (seqtype = 1) using the free ratio model (model = 2) with the transition/transversion ratio estimated from the data (fix_kappa = 0).

### Investigation of selection

The different tests for positive Darwinian selection are all based on extensions of the basic codon based likelihood model [11]. Likelihood ratio tests (LRTs) were used to compare nested models where one allows for positive selection and the other does not. The probability that a codon *i *substitutes into another codon *j *during the time interval t is determined by the rate matrix Q = (*q*_*ij*_) with entries


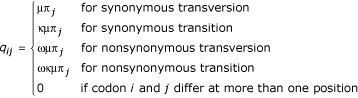


for *i *≠ *j*, with corresponding substitution probability matrix given by exp(Qt). Here π_*j *_is the equilibrium codon frequency of codon *j*, κ is the transition/transversion ratio and ω is the dN/dS ratio. All parameters are estimated independently for each gene. The star topology of the three species is used to estimate the branch lengths (τ_human_, τ_pig_, τ_mouse_) for synonymous and non-synonymous substitutions.

Positive selection was tested in two different ways. Test 1 averages over sites but differentiates among lineages. The LRT compares the free ratio model where all three lineages have a different value of ω estimated from the data with the one ratio model where all three lineages share a common value of ω [14]. We note that this test is more a test of variable dN/dS ratios among lineages than a test for positive selection. The free ratio model has three parameters for ω and the one ratio model only one. The LRT statistic is calculated as 2 times the differences in maximum log likelihood and is asymptotically distributed as a χ^2 ^distribution with 2 degrees of freedom. The genes found in one or more lineages evolving with a dN/dS ratio > 1 are compared to a nested model where the dN/dS ratio is fixed at 1 in the lineages shown to have a dN/dS ratio larger than one to see whether the result can be attributed to natural selection or just relaxation of selective pressures.

Test 2 is based on a new and improved version of the branch-site method presented in [23]. We will refer to this model as model A. The LRT is based on a comparison of the neutral model and model A. The neutral model assumes two categories of sites, a proportion *p*_1 _of sites where ω_1 _are estimated from the data and is forced to lie between 0 and 1, and a proportion p_2 _of neutrally evolving sites where ω_1 _= 1 (*p*_1 _+ *p*_2 _= 1). Model A furthermore allows a pre-specified branch to have a proportion of sites that evolve with a different value of ω estimated from the data. This value cannot be smaller than 1. The LRT follows a χ^2 ^distribution with 2 degrees of freedom. If the value of ω in the foreground lineage is estimated to be equal to one the model collapses to the neutral model.

PAML v. 3.14 [48] was used to estimate likelihood and parameters under each model. Codon equilibrium frequencies can be estimated from data using either simple proportions in the full data set (the CT model with 60 parameters), assuming equal frequencies (Fequal model), multiplying overall counts of nucleotide frequencies (F1 × 4 model, 3 parameters) or counts of nucleotide frequencies for each codon position (F3 × 4 model, 9 parameters). The codon table (CT) was used for analysis of the concatenated super gene and the F3 × 4 model was used on the individual genes.

### CpG Extension of the codon models

A simple extension of the F3 × 4 codon equilibrium frequency model can incorporate CpG avoidance by adding an extra parameter that penalizes a C followed by a G in the second and third codon position. The new model is parameterised as follows


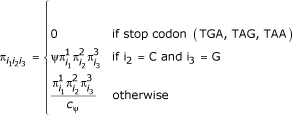


Here π_i_^1^_1 _represents the frequency of nucleotide *i*_1_, at codon position 1, and ψ(0 < ψ < 1) is a CpG penalizing parameter. The scaling factor c_ψ _ensures that the codon frequencies sum to one.

## Authors' contributions

FGJ carried out the analyses and was the primary writer of the text. AH and FGJ together implemented some of the analysis tools. FGJ, AH and MHS together developed the ideas and discussed the interpretation of the results. MF and CB gathered the EST data used. HHJ assembled the cDNAs and carried out Blast searches. All authors read and approved the final manuscript.

## Figures and Tables

**Table 1:** Overview of the codon models used in the analyses.

**Model**	**NP**	**Parameters**
*Lineage specific models*		
M_0_: One Ratio	5	κ, τ_pig_, τ_human_, τ_mouse_, ω
M_1a_: Free Ratio	7	κ, τ_pig_, τ_human_, τ_mouse_, ω_pig_, ω_human_, ω_mouse_
*Site specific models*		
M_1b_: Neutral	6	κ, τ_pig_, τ_human_, τ_mouse_, *p*_0 _(*p*_0 _+ *p*_1 _= 1), ω_[0;1[_
*Branch-Site specific models*		
M_2a_: Model A	8	κ, τ_pig_, τ_human_, τ_mouse_, *p*_0_(*p*_0 _+ *p*_1 _= 1), *p*_2, _ω_[0;1[, _ω_foreground_

The parameters used are (κ) transition / transversion ratio, (τ) branch length, (ω) dN/dS ratio, (p) fraction of codons that fall into the specified ω category.

**Table 2:** Comparison of topologies.

		**Log likelihood**	**Branch lengths**				
**Topology**	**No. genes**	**Super gene**	**Pig**	**Human**	**Mouse**	**Internal**	**Fugu**

*Amino Acids*							
A	245	-921354	0.0227	0.0280	0.0554	0.0083	0.3294
B	440	-920090	0.0292	0.0281	0.0403	0.0171	0.3229
C	180	-921703	0.0292	0.0241	0.0555	0.0055	0.3304
*1. codon pos.*							
A	215	-570181	0.0189	0.0235	0.0524	0.0088	0.2692
B	386	-568900	0.0265	0.0237	0.0341	0.0195	0.2600
C	208	-570504	0.0264	0.0190	0.0525	0.0058	0.2708
*2. codon pos.*							
A	215	-498689	0.0124	0.0156	0.0323	0.0053	0.1680
B	545	-498005	0.0167	0.0157	0.0229	0.0102	0.1642
C	175	-498925	0.0167	0.0130	0.0324	0.0034	0.1687

Top (A-C) refers to the three different topologies shown in Figure 3a–c. No. genes is the number of individual genes that favour each topology. The likelihood and the branch lengths shown are based on the concatenated super gene of the 988 individual four-species alignments; the average values of the branch lengths from the individual genes are highly similar to these results. Branch lengths are shown in number of substitutions per site.

**Table 3:** The rates of evolution.

	**Super gene**		**Synonymous substitutions**			**Nonsynonymous substitutions**		
	**dS**	**dN**	**Mean**	**Median**	**Variance**	**Mean**	**Median**	**Variance**

Human	0.115	0.017	0.126	0.118	0.003	0.018	0.010	0.0006
Pig	0.165	0.020	0.183	0.176	0.006	0.020	0.011	0.0005
Mouse	0.329	0.035	0.365	0.360	0.015	0.035	0.023	0.0013

Estimated rates of evolution on the super gene and the individual alignments. (dS) synonymous substitutions per codon, (dN) nonsynonymous substitutions per codon.

**Table 4:** Genes where all branches have ω > 1 based on the one ratio model.

		**Branch length**					**Number of substitutions**					
**Gene**	**L**	**Pig**	**Mouse**	**Human**	**Kappa**	**Omega**	**P(N)**	**P(S)**	**M(N)**	**M(S)**	**H(N)**	**H(S)**
NM_031268	72	0.096	0.102	0.128	2.844	1.481	5.7	1.3	6.0	1.3	7.6	1.6
NM_032353	97	0.105	0.269	0.104	3.183	1.206	7.6	2.6	19.5	6.6	7.5	2.6
XM_165930^a^	102	0.231	0.370	0.162	8.593	2.127	20.5	3.1	32.8	4.9	14.4	2.2
XM_168460^a^	74	0.176	0.527	0.132	2.665	1.121	8.7	4.3	26.1	12.9	6.5	3.2

Three-species alignments where the average dN/dS ratios over sites and lineages are larger than one. (Gene) Genbank accession number of the human gene. (L) Length of sequence alignment in codons, P(N) number of nonsynonymous substitutions in pig, P(S) number of synonymous substitutions in pig, M(N), M(S) and H(N), H(S) represents the mouse and human lineage respectively. (^a^)Possible pseudogene in human lineage.

**Table 5:** Genes with branches where ω > 1 based on the free ratio model.

		**Omega**			**Number of substitutions**						**Significance**
**Gene**	**L**	**Pig**	**Mouse**	**Human**	**P(N)**	**P(S)**	**M(N)**	**M(S)**	**H(N)**	**H(S)**	**ω > 1**

NM_001866	80	2.130	0.434	0.535	9.8	1.9	11.5	11.0	12.0	9.3	0.4775
NM_004085	97	1.088	0.053	0.000	2.0	0.5	4.9	24.1	0.0	8.8	0.9863
NM_004549	122	0.570	0.276	1.615	15.2	10.2	25.7	35.5	28.9	6.8	0.4229
NM_004891	65	0.092	0.116	2.560	2.9	11.6	8.1	25.1	10.1	1.4	0.5318
NM_006607	187	0.261	0.281	2.117	24.6	38.0	54.7	78.4	31.0	5.9	0.3625
NM_012198	216	0.341	0.073	1.849	24.9	26.5	18.3	91.6	29.4	5.8	0.3888
NM_017425	147	0.594	0.424	1.110	33.5	18.7	39.3	30.8	21.2	6.3	0.8750
NM_022978	60	0.228	0.143	1.102	2.8	4.4	3.3	8.0	38.9	12.5	0.8547
NM_031268	72	1.307	1.009	2.307	5.5	1.3	5.6	1.8	8.2	1.1	0.4009
NM_032353	97	0.539	2.100	0.964	5.71	4.3	22.0	4.3	7.1	3.0	0.1606
NM_032731	123	1.576	0.299	0.146	12.5	2.9	27.9	33.6	5.6	13.8	0.6854
XM_003044^a^	118	1.022	0.172	0.042	22.1	9.1	21.7	53.1	1.6	15.6	0.9743
XM_016532	155	0.000	0.125	1.193	0.0	34.0	16.0	49.0	11.4	3.6	0.8863
XM_041680^a^	168	0.415	0.186	1.079	29.4	25.5	33.1	64.0	6.7	2.2	0.9673
XM_062742^a^	110	0.000	0.011	1.661	0.0	19.0	1.0	36.4	22.9	5.4	0.5119
XM_069411	187	0.085	0.058	1.108	3.7	15.9	5.5	34.9	119.9	39.6	0.6943
XM_092681	81	0.187	0.040	1.167	5.3	10.0	3.0	26.0	15.9	4.8	0.8551
XM_165930^a^	102	∞	2.061	0.691	24.7	0.0	32.9	5.1	10.2	4.7	0.0020*
XM_166695^a^	190	0.235	0.067	1.183	19.8	30.9	12.5	38.3	79.6	24.7	0.6657
XM_168460^a^	74	2.672	0.848	1.085	10.4	2.3	23.9	15.6	6.6	3.4	0.9234
XM_172026^a^	72	0.035	0.042	2.213	0.9	8.8	2.1	17.3	30.1	4.7	0.2564
XM_172342^a^	143	0.000	0.032	1.320	0.0	3.0	2.0	20.3	27.3	6.6	0.6075
XM_172363^a^	77	0.139	0.132	1.077	8.7	21.0	8.6	22.0	14.4	4.5	0.9422

Three-species alignments where one or more lineages have a dN/dS ratio larger than one. (Gene) Genbank accession number of the human gene. (L) Length of sequence alignment in codons, P(N) number of nonsynonymous substitutions in pig, P(S) number of synonymous substitutions in pig, M(N), M(S) and H(N), H(S) represents the mouse and human lineage respectively. (^a^)Possible pseudogenes in human lineage. (ω > 1) If more than one branch have ω > 1 only the significance of the branch with the largest value of ω is shown. (*) LRT (ω > 1) significant at 0.01 level.

**Table 6:** Genes predicted to be under positive selection with the branch-site models.

**Genbank Acc.**	**ω**	**P**	**Human gene annotation**
*Human lineage*			
NM_001785	3.8	0.27	Cytidine deaminase
NM_001867	5.3	0.20	Cytochrome c oxidase subunit VIIc
NM_004846	10.7	0.05	Eukaryotic translation initiation factor 4E-like 3
NM_006607	27.9	0.23	Pituitary tumor-transforming 2
NM_012198	4.8	0.32	Grancalcin, EF-hand calcium binding protein
NM_021167	6.9	0.13	Ocular development-associated gene (Interim)
NM_022978	22.0	0.28	Small EDRK-rich factor 1B (centromeric)
NM_080915	28.1	0.12	Deoxyguanosine kinase
XM_039644	49.5	0.02	Unclassified
XM_059906	8.2	0.07	Unclassified
XM_062742	2.0	0.84	Unclassified
XM_069411	6.8	0.38	Similar to RIKEN cDNA 1300003K24 (Interim)
XM_166695	6.9	0.31	Unclassified
XM_167131	3.8	0.28	Unclassified
XM_172026	16.9	0.38	Unclassified
*Pig lineage*			
NM_000509	2.4	0.13	Fibrinogen, gamma polypeptide
NM_000520	10.3	0.06	Hexosaminidase A (alpha polypeptide)
NM_002979	2.0	0.15	Sterol carrier protein 2
NM_003142	3.1	0.14	Sjogren syndrome antigen B (autoantigen La) *
NM_016489	2.4	0.32	5'-nucleotidase, cytosolic III
*Mouse lineage*			
NM_005731	8.6	0.074	Actin related protein 2/3 complex, subunit 2, 34 kDa
NM_013342	1.3	0.152	TCF3 (E2A) fusion partner (in childhood leukaemia)
NM_023935	3.0	0.171	Chromosome 20 open reading frame 116
XM_007076	2.5	0.248	Unclassified

Genes shown here all have a significant LRT at the 0.001 level. (ω) the predicted dN/dS ratio in the foreground lineage. (p) the proportion of sites predicted to be under positive selection.

**Table 7:** Heterogeneity in dN/dS ratios over sites.

	**Fraction of codons**					**Branchlength**			
**Model**	**ω = 0**	**ω = 1/3**	**ω = 2/3**	**ω = 1**	**ω = 3**	**Pig**	**Mouse**	**Human**	**Kappa**

CT	0.691	0.238	0.055	8 × 10^-5^	0.016	0.221	0.418	0.163	2.494
F3 × 4	0.681	0.245	0.058	1 × 10^-5^	0.017	0.219	0.410	0.162	2.658

The concatenated super gene is used to estimate the distribution of dN/dS ratios over sites. Each codon is allowed to fall into one of the five predefined dN/dS ratio classes. The branch lengths are expressed as number of substitutions per codon.

**Table 8:** Codon usage in the three mammalian species.

	**Frequency**				**Frequency**				**Frequency**				**Frequency**		
**Codon**	**H**	**M**	**P**	**Codon**	**H**	**M**	**P**	**Codon**	**H**	**M**	**P**	**Codon**	**H**	**M**	**P**

**TTT(F)**	2.16	2.00	2.06	**TCT(S)**	1.43	1.44	1.38	**TAT(Y)**	1.61	1.40	1.48	**TGT(C)**	1.03	0.99	0.97
**TTC**	1.85	2.01	1.93	**TCC**	1.30	1.40	1.37	**TAC**	1.48	1.64	1.60	**TGC **	0.92	0.97	0.96
**TTA(L)**	0.98	0.81	0.89	**TCA**	1.12	1.01	1.04	TAA(*)	0	0	0	TGA(*)	0	0	0
**TTG**	1.50	1.42	1.46	**TCG**	**0.33**	**0.39**	**0.40**	TAG(*)	0	0	0	**TGG(W)**	1.24	1.23	1.23
**CTT**	1.53	1.40	1.44	**CCT(P)**	1.66	1.62	1.60	**CAT(H)**	1.17	1.05	1.07	** CGT(R)**	**0.53**	**0.49**	**0.51**
**CTC**	1.55	1.67	1.70	**CCC**	1.35	1.37	1.48	**CAC**	1.15	1.31	1.25	**CGC **	**0.79**	**0.80**	**0.83**
**CTA**	0.83	0.78	0.74	**CCA**	1.63	1.58	1.53	**CAA(Q)**	1.30	1.18	1.19	**CGA **	**0.75**	**0.79**	**0.76**
**CTG**	3.32	3.60	3.48	**CCG**	**0.47**	**0.55**	**0.54**	**CAG**	3.03	3.17	3.13	**CGG **	**0.99**	**1.02**	**1.06**
**ATT(I)**	2.16	1.90	2.01	**ACT(T)**	1.42	1.30	1.32	**AAT(N)**	2.05	1.73	1.88	**AGT(S)**	1.18	1.12	1.13
**ATC**	2.06	2.24	2.20	**ACC**	1.53	1.61	1.62	**AAC**	1.76	1.99	1.86	**AGC **	1.39	1.52	1.44
**ATA**	0.92	0.84	0.88	**ACA**	1.64	1.59	1.50	**AAA(K)**	3.35	2.95	3.23	**AGA(R)**	1.46	1.40	1.42
**ATG(M)**	2.22	2.20	2.22	**ACG**	**0.47**	**0.56**	**0.58**	**AAG**	3.74	3.98	3.65	**AGG **	1.04	1.14	1.08
**GTT(V)**	1.49	1.37	1.41	**GCT(A)**	2.21	2.22	2.11	**GAT(D)**	2.93	2.59	2.79	** GGT(G)**	1.32	1.23	1.26
**GTC**	1.33	1.50	1.43	**GCC**	2.35	2.43	2.54	**GAC**	2.35	2.70	2.51	**GGC **	1.95	2.08	0.06
**GTA**	0.99	0.84	0.88	**GCA**	1.89	1.77	1.81	**GAA(E)**	3.77	3.44	3.62	**GGA **	2.05	1.95	1.97
**GTG**	2.66	2.82	2.75	**GCG**	**0.63**	**0.75**	**0.73**	**GAG**	3.51	3.79	3.66	**GGG **	1.28	1.35	1.34

The frequencies are expressed as a percentage of the 240,048 codons in each of the three species. Human(H), Mouse(M), Pig(P). Stop codons are not allowed in the analyses (*).

**Table 9:** Evaluation of the choice of codon equilibrium frequencies.

		**Estimated Branch Lengths**						
**Model**	**df**	**Human**	**Mouse**	**Pig**	κ	ω	**lnL**	**X^2^**

FQ	1	0.136	0.340	0.178	2.862	0.125	-1502578	249354
F1 × 4	3	0.134	0.335	0.175	2.776	0.122	-1500436	231560
F3 × 4	9	0.136	0.343	0.178	2.692	0.119	-1495232	133363
F3 × 4 + CpG	10	0.138	0.351	0.181	2.593	0.114	-1493996	74214
Codon Table	60	0.136	0.348	0.179	2.497	0.113	-1478877	0

The values are estimated with the concanated gene comprising 240,048 codons using the one ratio model. (df) Number of parameters (κ) Transition/transversion ratio. (ω) dN/dS ratio. (X^2^) A chi square test statistic comparing the expected frequencies of each codon to the observed codon counts.
